# Draft Genome Sequences of Two Bacteriocin-Producing Enterococcus faecium Strains Isolated from Nonfermented Animal Foods in Spain

**DOI:** 10.1128/mra.00866-22

**Published:** 2022-10-12

**Authors:** Marcos Quintela-Baluja, Kelly Jobling, David W. Graham, Mohamed Alnakip, Karola Böhme, Jorge Barros-Velázquez, Pilar Calo-Mata

**Affiliations:** a Department of Analytical Chemistry, Nutrition and Food Science, School of Veterinary Sciences, University of Santiago de Compostela, Lugo, Spain; b School of Engineering, Newcastle University, Newcastle upon Tyne, United Kingdom; c Department of Food Control, Faculty of Veterinary Medicine, Zagazig University, Al Sharqiyah, Egypt; Indiana University, Bloomington

## Abstract

Here, we report the draft genome sequences of two bacteriocin-producing Enterococcus faecium strains isolated from nonfermented animal foods in Spain. The genomes of the strains contain at least three different regions encoding bacteriocins, and the strains comply with the European Food Safety Authority guidance for use in animal nutrition.

## ANNOUNCEMENT

*Enterococcus* is a controversial genus of Gram-positive lactic acid bacteria that includes pathogenic, spoilage, and harmless strains (1). Enterococcus faecium represents the most important enterococcus in food fermentation and spoilage, and its use has also been reported as a probiotic for more than 2 decades without any adverse effects (1, 2). On the other hand, E. faecium has rapidly evolved as a worldwide nosocomial pathogen (2), and this raises questions about its safety for use in foods or as probiotics.

Here, we present the genome of two bacteriocin-producing *Enterococcus* strains previously isolated from refrigerated vacuum-packaged beef in Spain (LHICA_28.4 and LHICA_40.4) (3). The strains inhibited the growth of four relevant foodborne pathogenic and spoilage bacteria and were able to survive down to pH 3.0 in the presence of bile salts, pancreatin, and pepsin (4). They also showed good adhesion properties and sensitivity to clinically relevant antimicrobial agents (4). The sequencing of the 16S rRNA gene identified them as Enterococcus faecium, and a PCR screening for class IIa bacteriocins detected the presence of the enterocin P gene (3).

Bacterial isolates were grown from stocks in De Man, Rogosa and Sharpe (MRS) broth at 37°C overnight (Oxoid, UK) and plated on MRS agar (Oxoid, UK) at 37°C overnight. One bacterial colony was picked and streaked on a new MRS plate. Then, biomass from each strain was harvested and resuspended in Microbank beads (Pro-Lab Diagnostics, UK) and sent to MicrobesNG (Birmingham, UK) for genomic DNA extraction and sequencing. Briefly, three beads were washed with DNA extraction buffer containing lysozyme and RNase A and incubated for 25 min at 37°C. Proteinase K and RNase A were added, and mixtures were incubated for 5 min at 65°C. Genomic DNA was purified using an equal volume of solid-phase reversible immobilization beads (ABM, Canada) and resuspended in EB buffer (10 mM Tris-Cl [pH 8.5]). Genomic DNA libraries were prepared using a Nextera XT library prep kit (Illumina, USA) following the manufacturer's protocol. The pooled libraries were quantified using the Kapa Biosystems library quantification kit for Illumina and sequenced on the Illumina HiSeq 2500 instrument using a 250-bp paired-end protocol. The reads were trimmed using Trimmomatic version 0.39 (5) with a sliding window quality cutoff of Q15. Sequence reads were assembled into contigs using SPAdes version 3.7 (6). The two assemblies were then annotated using the NCBI Prokaryotic Genome Annotation Pipeline v5.3 (7). The sequencing and assembly metrics of the two genome assemblies are summarized in Table 1. Following genome annotation, the draft genomes were analyzed using BAGEL4 to search for potential antimicrobial-encoding operons (8). Default settings were used for all software unless otherwise specified.

**TABLE 1:** Sequencing and assembly metrics of E. faecium strains LHICA_28.4 and LHICA_40.4

Genome feature	Value(s)	
	LHICA_28.4	LHICA_40.4
Sequencing coverage (×)	137	101
Assembly size (bp)	2,772,417	2,783,548
No. of contigs (>1,000 bp)	148	99
Largest contig (bp)	147,223	157,844
*N*_50_ (bp)	34,752	67,635
*L*_50_ (contigs)	24	14
GC content (%)	38.04	37.85
Total no. of genes	2,872	2,882
Total no. of CDSs^*a*^	2,794	2,798
Total no. of coding genes	2,649	2,605
No. of rRNAs (5S, 16S, 23S)	4, 3, 3	3, 6, 7
No. of complete rRNAs (5S, 16S, 23S)	3, 1, 1	3, —, —
No. of predicted tRNAs	64	84
No. of predicted noncoding RNAs	4	4
No. of pseudogenes	145	193

a CDSs, coding DNA sequences.

These data provide insights into the genetic basis of bacteriocin-producing E. faecium from nonfermented animal foods. Furthermore, the studied strains have the potential to produce bacteriocins that might be employed as biopreservatives. Thus, each strain codes for at least three different bacteriocins, and they comply with the European Food Safety Authority guidance for distinguishing between safe and potentially harmful strains of E. faecium in animal nutrition (9).

### Data availability.

The raw sequencing data are available at the NCBI Sequence Read Archive under BioProject PRJNA863767 with accession numbers SRX16741203 (LHICA_28.4) and SRX16741202 (LHICA_40.4). The draft genome sequences have been deposited in GenBank under the accession numbers JANIND000000000 (LHICA_28.4) and JANINE000000000 (LHICA_40.4). The versions described in this paper are JANIND000000000.1 and JANINE000000000.1.
